# Spermatic Cord Metastasis of Primary Hepatocellular Carcinoma Presenting as an Inguinal Mass: A Case Report

**DOI:** 10.5402/2011/612753

**Published:** 2011-04-07

**Authors:** Heng-Chieh Chiang, Pao-Hwa Chen, Hung-Jen Shih

**Affiliations:** Division of Urology, Department of Surgery, Changhua Christian Hospital, 135 Nanxiao Street, Changhua City, Changhua County 500, Taiwan

## Abstract

Most spermatic cord masses are benign, and malignant spermatic cord tumors are uncommon. Spermatic cord metastases originating from hepatocellular carcinoma (HCC) have not been previously reported in the English language literature as determined by a PubMed search. We report a male patient who presented with a painful palpable mass in the right inguinal area. The patient was diagnosed with HCC in 2004 and undertook a nonsurgical approach to control the cancer. A radical orchiectomy was performed, and the pathological report showed metastatic HCC in the spermatic cord. The patient received palliative radiation therapy because of a positive surgical margin. No recurrence was noted after 6 months of followup.

## 1. Introduction

Extrahepatic lesions are not uncommon in late-stage HCC patients. These extrahepatic lesions are most commonly found in the lungs, lymph nodes, and bones [1]. There have been reports of extrahepatic lesions found in urogenital sites, such as the kidneys, testes, and bladder [2–4]. We encountered a patient with a metastatic HCC lesion presenting as a tender right inguinal mass. To the best of our knowledge, this case represents the first such report in the English language literature.

## 2. Case Report

A 57-year-old man was referred to our urology out-patient department from a gastrointestinal doctor because of a tender inguinal mass that had become progressively larger over the previous one and half months. The patient had a past history of HCC (underwent radiofrequency ablation in July 2004 and transcatheter arterial chemoembolization in November 2004), hepatitis B virus infection, rightside inguinal hernia (status postherniorrhaphy in 2007), a cholecystectomy in 2000, and a vasectomy in the 1990s.

**Figure 1:**
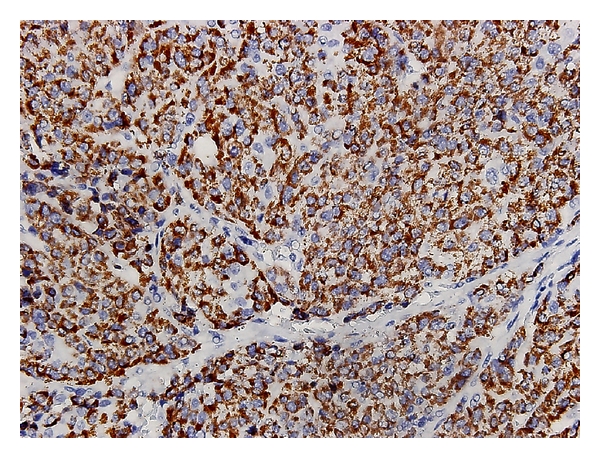
Metastatic HCC showed diffuse immunoreactivity of Hep Par 1 (H&E, ×400).

**Figure 2:**
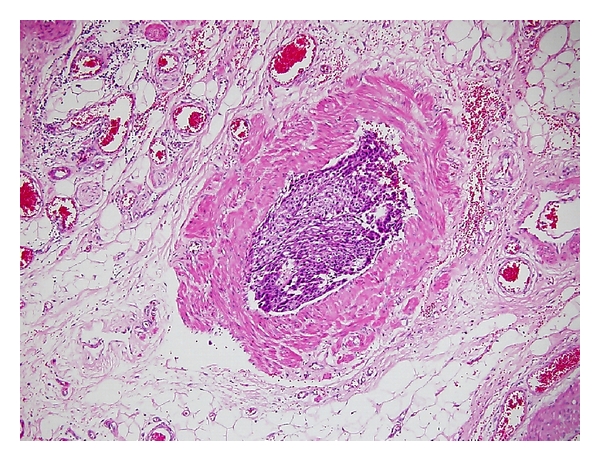
The metastatic tumor emboli from the HCC in the spermatic cord vessel (H&E, ×100).

He noticed a painful hard mass in the right inguinal area one and half months prior to visiting our department. The mass was progressively enlarging and was nonreducible. Physical examination revealed a 2 × 2 centimeter hard, movable, tender mass in the right inguinal area. An excision biopsy was performed, and the pathological report revealed tumor cells with abundant amphophilic to pale eosinophilic cytoplasm, round nuclei, mitosis, and focal necrosis, and the cells were Hep Par 1 positive as determined by immunohistochemical staining (Figure 1). The diagnosis of metastatic HCC to the spermatic cord was made. Due to a persistent, painful sensation in the right inguinal area, removal of the right inguinal metastatic mass was arranged. During inguinal exploration, it was observed that the tumor wrapped around the spermatic cord. A right radical orchiectomy was performed because the tumor could not be freed from the spermatic cord. The pathological report showed multiple metastatic HCC tumor nodules and tumor emboli in the lymphatic and blood vessels of the spermatic cord and peritesticular soft tissue (Figure 2). The testes and epididymis were not involved in the tumor. The resection margin of the spermatic cord included tumor emboli. Palliative radiation therapy was administered to the tumor bed after the wound had healed. The total dose of radiation therapy is 4500 centigray divided into 25 fractions. There was no tumor recurrence after 6 months of followup.

## 3. Discussion

HCC is the most common primary malignant tumor of the liver. HCC is commonly seen in Taiwanese hospitals due to high incidence of hepatitis B and C in Taiwan. Extrahepatic metastases of hepatocellular carcinoma are not uncommon in late-stage HCC patients. Most documented extrahepatic metastatic lesions have been found in the lungs followed by the lymph nodes, bones, and adrenal glands, but there are cases of distal metastasis to the genitourinary system, such as the kidneys and bladder [1, 3, 4]. Similar to other metastatic lesions, the clinical presentation of extrahepatic metastatic lesions depends on their location and the affected areas. Presentations include hematuria, flank pain, and testicular pain; these signs and symptoms are very similar to those of other common genitourinary diseases [1–4]. In our case, this patient presented with a painful inguinal mass. 

When patients present with an inguinal mass, we need to take into consideration several differential diagnoses ranging from benign to malignant lesions [5]. Spermatic cord tumors are rare. Malignant tumors, including primary tumors and metastases, are extremely rare [6–8]. Painless scrotal or lower inguinal mass was the most common clinical presentation in the metastatic spermatic cord tumors [6]. The metastatic spermatic cord tumors could be misdiagnosed as hydrocele, hernia, and testis tumor [9]. When a tumor is found in the inguinal area in patients with a history of malignancy or peritoneal carcinomatosis, a diagnosis of a metastatic mass is to be considered first [5]. What are the possible mechanisms of malignant tumor cells spread to the spermatic cord? The two primary ways to the spermatic cord are hematogenous and lymphatic dissemination. Retrograde extension through the vas deference and transperitoneal seeding had been reported [10]. 

In this case, a metastatic mass was highly suspected due to the history of HCC. A metastatic tumor originating from HCC was confirmed after excision biopsy. According to the national comprehensive cancer network clinical practice guideline for metastatic HCC [11], only sorafenib (for Child-Pugh Class A or B), supportive care, or clinical trials are recommended therapies. Because of persistent pain in the inguinal area and progressive enlargement of the tumor, a radical orchiectomy was performed. 

 What kind of treatment is suitable for tumor emboli in the surgical margin of the spermatic cord? According to previous reports, palliative radiation therapy has been used to treat HCC with brain and bone metastasis with successful symptom control [12]. Radiation therapy is effective for the local control of lymph node metastasis from HCC [13]. Hawkins and Dawson showed that radiation therapy can prolong survival for HCC patients with portal vein thrombus [12]. Because of the lack of a standard treatment for metastatic lesions of HCC, we used radiation therapy for local control, and no recurrence was found after 6 months. 

In conclusion, we report the first case of metastatic HCC in the spermatic cord, which presented as a painful inguinal mass. Complete tumor excision with clear margins should be performed for spermatic cord tumors. Palliative radiation therapy is a choice for the treatment of metastatic HCC with tumor emboli in the surgical margin.
